# Molecular Docking-Based Virtual Screening of FDA-Approved Drugs Using Trypanothione Reductase Identified New Trypanocidal Agents

**DOI:** 10.3390/molecules29163796

**Published:** 2024-08-10

**Authors:** Rogelio Gómez-Escobedo, Domingo Méndez-Álvarez, Citlali Vázquez, Emma Saavedra, Karina Vázquez, Verónica Alcántara-Farfán, Joaquín Cordero-Martínez, Alonzo Gonzalez-Gonzalez, Gildardo Rivera, Benjamín Nogueda-Torres

**Affiliations:** 1 Departamento de Parasitología, Escuela Nacional de Ciencias Biológicas, Instituto Politécnico Nacional, Ciudad de México 07738, Mexico; rogelio.gomez.14@gmail.com; 2 Laboratorio de Biotecnología Farmacéutica, Centro de Biotecnología Genómica, Instituto Politécnico Nacional, Reynosa 88710, Mexico; doomadv@hotmail.com (D.M.-Á.); agonzalezg1700@alumno.ipn.mx (A.G.-G.); 3 Departamento de Bioquímica, Instituto Nacional de Cardiología Ignacio Chávez, Ciudad de México 14080, Mexico; cita2812@gmail.com (C.V.); emma_saavedra2002@yahoo.com (E.S.); 4 Facultad de Medicina Veterinaria y Zootecnia, Universidad Autónoma de Nuevo León, General Escobedo 66050, Mexico; kwvazque@gmail.com; 5 Laboratorio de Bioquímica Farmacológica, Escuela Nacional de Ciencias Biológicas, Instituto Politécnico Nacional, Ciudad de México 07738, Mexico; valcantaraf@ipn.mx (V.A.-F.); jcorderom@ipn.mx (J.C.-M.)

**Keywords:** Chagas disease, trypanothione reductase, molecular docking, repositioning, FDA drugs

## Abstract

American trypanosomiasis or Chagas disease, caused by *Trypanosoma cruzi* (*T. cruzi*), affects approximately 6–7 million people worldwide. However, its pharmacological treatment causes several uncomfortable side effects, causing patients’ treatment abandonment. Therefore, there is a need for new and better treatments. In this work, the molecular docking of nine hundred twenty-four FDA-approved drugs on three different sites of trypanothione reductase of *T. cruzi* (*Tc*TR) was carried out to find potential trypanocidal agents. Finally, biological evaluations in vitro and in vivo were conducted with the selected FDA-approved drugs. Digoxin, alendronate, flucytosine, and dihydroergotamine showed better trypanocidal activity than the reference drugs benznidazole and nifurtimox in the in vitro evaluation against the trypomastigotes form. Further, these FDA-approved drugs were able to reduce 20–50% parasitemia in a short time in an in vivo model, although with less efficiency than benznidazole. Therefore, the results suggest a combined therapy of repurposed and canonical drugs against *T. cruzi* infection.

## 1. Introduction

Chagas disease, also known as American trypanosomiasis, is an anthropozoonotic disease caused by the *Trypanosoma cruzi* (*T. cruzi*) parasite, which is acquired mainly through the feces of hematophagous Hemiptera vectors of the Triatomine subfamily. Other ways of contracting the parasite are congenital transmission, blood transfusions, organ transplants, oral transmission by food contaminated with infected feces, and laboratory accidents. It is estimated that Chagas Disease affects approximately 6–7 million people, mainly in Latin America and Caribbean endemic countries, although cases in non-endemic countries have been documented due to human immigration [1,2]. Chagas disease presents three phases: the acute phase, with a duration of 2–8 weeks with high parasitemia levels but with nonspecific signs and symptoms; the indetermined phase, with no signs or symptoms, where two-thirds of those infected remain in this phase; and the chronic phase, where the remaining third progresses to and where cardiopathies such as arrhythmias, septal defects, cardiac failure, and death occur [3].

At present, there are only two drugs for its treatment, nifurtimox (Nfx) and benznidazole (Bzn), which have shown a moderately parasitological cure in the acute phase but are ineffective in the chronic phase. In addition to the latter, the long periods of treatment and their severe side effects cause people to abandon the treatment, which means that the parasitological cure is not achieved, with the possibility of the emergence of variants that are resistant to these drugs. Therefore, new drugs are required for the treatment of the disease [4].

In the search for new drugs, targeted drug repositioning is a strategy that has been used in the last decades to obtain new trypanocidal agents; the use of this strategy allows for reducing time and costs in the development and search for treatments against existing diseases [5]. Different approaches have been tested to reposition drugs for the treatment of Chagas disease, the most common being through in silico studies. In this approach, molecular docking on a directed target has been a very useful tool that has allowed for the virtual screening of huge libraries of compounds; the strategy has also permitted the establishment of predictions about the potential ways in which drugs/compounds are able to inhibit enzymes and to understand the molecular interactions that exists between them [6].

Trypanothione reductase (TR) is an enzyme that is central in the trypanothione-dependent redox system of the parasite, which indirectly participates in the detoxification of reactive oxygen species (ROS) that the parasite must deal with once it is inside the mammalian host. This redox system involves the utilization of NADPH reducing equivalents that are fed into a variety of enzymatic detoxification pathways utilizing trypanothione as a vehicle. Trypanothione is oxidized as it yields its electrons into the detoxification paths, later it is then reduced by TR, and it may be now recycled [7]. This enzyme represents an attractive pharmacological target since it is not found in the mammalian host and its identity with human glutathione reductase (which is its analogue) is low, so even inhibiting the parasite enzyme, the host enzyme will not be affected. There are three sites of interest: the Z site, mepacrine, and the catalytic site. The Z site is a hydrophobic barrier located in the proximity of the catalytic site, deep within the interface cavity, in close vicinity to the NADPH binding site; in previous studies, it has been mentioned that by binding at this Z site, it is possible to sequester the catalytic site of TR through non-competitive inhibition [8]. The mepacrine site is a hydrophobic region located at the entrance of the trypanothione disulfide binding site; this site is where most inhibitors bind, acting through competitive inhibition. The catalytic site, the place where the reduction of trypanothione disulfide takes place, was chosen to inhibit through competitive inhibition [9]. The catalytic triad is composed of Cys53, Cys58, and His461.

In this study, a molecular docking-based virtual screening of FDA-approved drugs using the enzyme TR of *T. cruzi* (*Tc*TR) as a pharmacological target was performed centered at each of these three sites to identify new agents with potential trypanocidal activity. Finally, biological in vitro and in vivo evaluations against trypomastigotes from *T. cruzi* NINOA and INC-5 strains were carried out with the selected FDA-approved drugs.

## 2. Results

### 2.1. Binding Sites

Nine hundred twenty-four FDA-approved drugs were evaluated by molecular docking on three distinct sites of the *Tc*TR enzyme: (a) Z site composed of three residues, Phe396, Pro398, and Leu399, is a hydrophobic region present in the neighborhood of the catalytic site and the NADPH binding site [10]; (b) mepacrine site is a hydrophobic region found at the entrance of the trypanothione disulfide binding cavity, the most prominent residues in this site are Trp21 and Met113, and this site takes relevance since most of the inhibitors that have been tested for this enzyme bind here, thus providing competitive inhibition [9]; and (c) catalytic site, where the reduction of trypanothione disulfide takes place, was selected to search for competitive inhibitors, as the residues involved with interactions at this site are mainly His461, Glu466, and Glu467 [9]. The results of the top 20 FDA-approved drugs according to docking score values for each of the docking sites are shown in Table 1. The most promising interaction profiles were constructed from the docking of several known TR inhibitors at each of the docking sites; the full results may be consulted in the Supplementary Materials.

### 2.2. Molecular Docking on Z Site

Molecular docking on the Z site of the nine hundred twenty-four FDA-approved drugs allowed us to obtain the binding energy value of each drug. The top 20 are shown in Table 1; these compounds have docking scores (DS) ranging from −8.864 to −10.148 kcal/mol, where most are comparable to control inhibitors tested. The control inhibitors ZINC12151998, 7i, and 7e (previously reported as enzyme inhibitors at the Z site) had binding energy values of −10.3, −9.838, and −9.428 kcal/mol, respectively.

**Figure 1:**
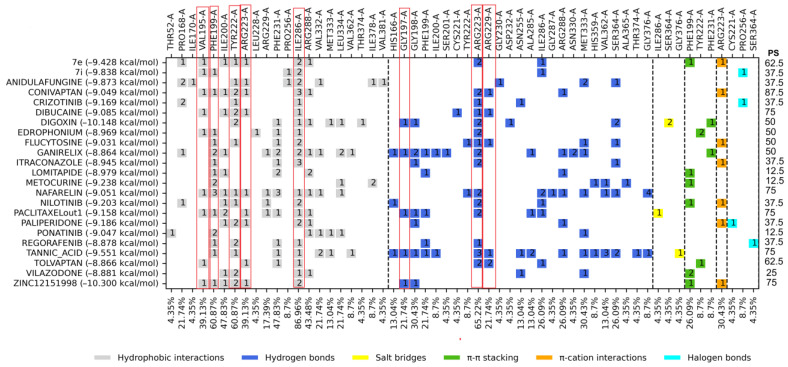
Interaction profiles for the top 20 FDA-approved drugs docked and centered at the Z site. PS: The percentage of similarity; red boxes highlight the eight amino acid residues that are part of the observed consensus profile in docked inhibitors (full information in Supplementary Materials). Dashed lines separate between interaction types, and the number inside the colored squares indicates the number of interactions for that residue with the ligand.

Subsequently, an analysis of the ligand–protein interactions of the top 20 FDA-approved drugs and control compounds was performed. The profile with hydrophobic interactions (HI), hydrogen bonds (HB), ππ–cation interactions (π-c), ππ–π stacking (π-s), halogen interactions (HalB), and salt bridges (SB) is shown in Figure 1. As the reported Z site is a triad of amino acid residues deep within the cavity of the interface, all the calculated docking grid boxes encompass the NADPH binding site as well. The NADPH binding site resides in the vicinity of the catalytic site given its function as the source of reducing equivalents; thus, docking in this deep portion of the cavity resulted in a dual site docking. The interacting residues presented in Figure 1 occur mostly within the NADPH cavity, related to residues that are part of the binding of the natural ligand NADPH: Gly197, Gly198, Phe199, Tyr222, Arg223, Arg229, Asn255, and Ile286.

### 2.3. Molecular Docking on Mepacrine Site

**Figure 2:**
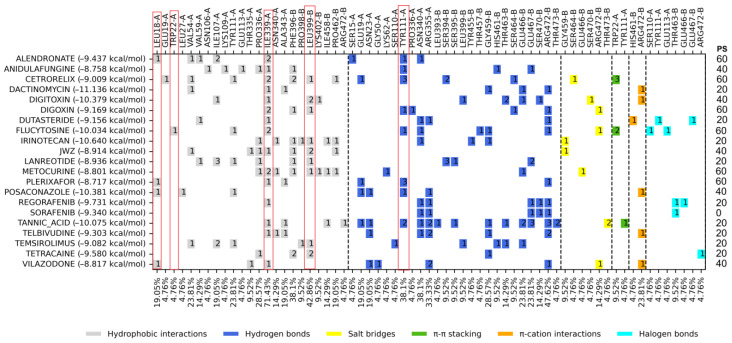
Interaction profiles for the top 20 FDA-approved drugs docked and centered at the mepacrine site. PS: The percentage of similarity; red boxes highlight the five amino acid residues that are part of the observed consensus profile in docked inhibitors (full information in Supplementary Materials). Dashed lines separate between interaction types, and the number inside the colored squares indicates the number of interactions for that residue with the ligand.

Of the nine hundred twenty-four FDA drugs evaluated by molecular docking on the mepacrine active site, the top 20 FDA-approved drugs with the best binding energy were obtained, with DS values ranging from −8.717 to −11.136 kcal/mol, where most are better than the control compound JWZ. The control compound JWZ had a binding energy of −8.914 kcal/mol (Table 1). The analysis of the ligand–protein interaction profile is shown in Figure 2.

### 2.4. Molecular Docking on the Catalytic Site

**Figure 3:**
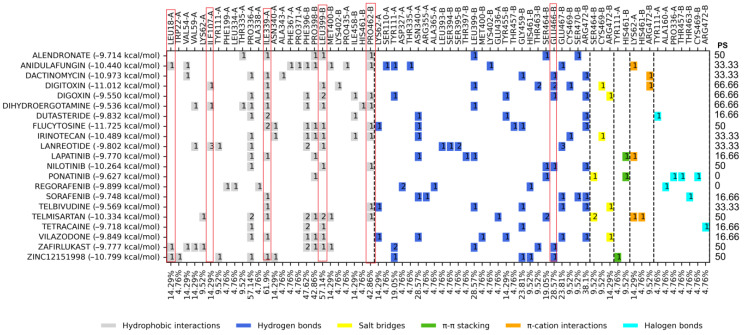
Interaction profiles for the top 20 FDA-approved drugs docked centered at the catalytic site. PS: Percentage of similarity, red boxes highlight the six amino acid residues that are part of the observed consensus profile in docked inhibitors (full information in Supplementary Materials). Dashed lines separate between interaction types, and the number inside the colored squares indicates the number of interactions for that residue with the ligand.

Like the previous binding sites, the top 20 FDA-approved drugs with the best binding energy on the catalytic site were selected with DS values ranging from −9.536 to −11.725 kcal/mol. The control compound ZINC12151998 presented a binding energy of −10.799 kcal/mol (Table 1). The analysis of the ligand–protein interaction profile is shown in Figure 3.

### 2.5. Molecular Dynamic Analysis

A molecular dynamic analysis was performed for alendronate (at mepacrine site), digoxin (at Z-site), dihydroergotamine (at catalytic site), and flucytosine (at mepacrine site), the four compounds chosen to be further tested as trypanocidal agents to determine the ligand–*Tc*TR complex stability; the dynamics were analyzed with three measurements: RMSD, RMSF, and the radius of gyration.

#### 2.5.1. Root Mean Square Deviation (RMSD) Analysis

**Figure 4:**
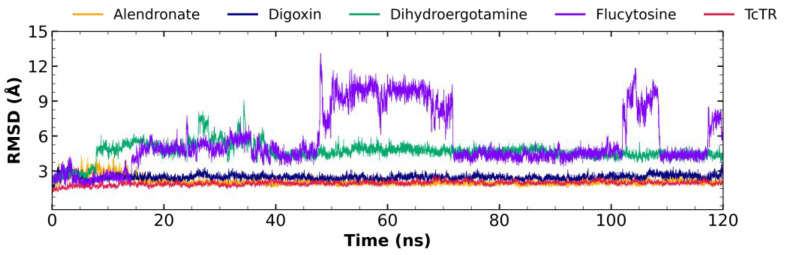
RMSD graph for fluctuations over time for *Tc*TR complex fluctuation: alendronate (1.25–4.31 Å), digoxin (1.15–3.72 Å), dihydroergotamine (0.98–9.06 Å), flucytosine (1.08–13.1 Å), and *Tc*TR (0.33–2.22 Å).

Figure 4 shows the RMDS fluctuations for the alendronate–*Tc*TR complex (orange), digoxin–*Tc*TR (blue), dihydroergotamine–*Tc*TR (green), flucytosine–*Tc*TR (violet), and free *Tc*TR (red). The alendronate–*Tc*TR complex shows a maximum fluctuation of 4.31 Å; still, most of the dynamics maintain a fluctuation in the range of 2–3 Å, with a mean of 2.09 ± 0.33 Å, showing stable behavior throughout most of the 120 ns trajectory. The digoxin–*Tc*TR complex shows a maximum fluctuation of 3.72 Å; still, most of the dynamics maintain a fluctuation in the range of 2–3 Å, with a mean of 2.47 ± 0.23 Å, showing stable behavior throughout most of the 120 ns trajectory. The dihydroergotamine–*Tc*TR complex shows a maximum fluctuation of 9.06 Å, which may be observed at about 35 ns; still, the dynamics remain mostly stable after the first 40 Å, with a fluctuation around 4.5 Å, and the overall fluctuation has a mean of 4.66 ± 0.78 Å, while for the plateau-like portion of the graph, it has a fluctuation with a mean of 4.58 ± 0.32 Å. On the other hand, flucytosine–*Tc*TR is the least stable complex, as it shows a fluctuating behavior throughout except for two plateau-like portions at 72–100 ns and 112–116 ns; the overall average RMSD is 5.61 ± 2.40 Å, while for these two short lapses, it holds an average RMSD of 4.39 ± 0.34 Å and 4.36 ± 0.25 Å, respectively.

Ten evenly spaced frames were extracted from each nanosecond trajectory (every 100 ps) for the second half of the trajectory (60–120 ns), where stability was observed for the RMSD plot, and these were analyzed to determine their interaction profiles, which, altogether, were 600 frames. It was observed that for alendronate, the top five most frequently occurring interactions were Ile339 (HI: 95.67%), Val54 (HI: 78.70%), Pro336 (HI: 77.54%), and Val59 (HB: 63.89%), and it may be added that two less frequent, but important interactions occur with His461 (HI: 59.07%) and Glu466 (HI: 57.90%). For the analysis of the interaction profile, it is observed that digoxin maintains a stable binding mode (over 90% observed frequency) with a relatively high number of residues, that is 7, five of these through a strong type of interaction (HB and SB), His428 (SB: 99.67%), Gly198 (HB: 98.34%), Phe199 (HI: 96.01%), Asp232 (HB: 95.02%), Ala285 (HB: 94.52%), Ile286 (HB: 94.35%), and Phe231 (HI: 92.69%); additionally, two are less frequent but are relevant to NADPH binding Arg229 (SB:88.87%) and Gly197 (HB: 76.58%). As for dihydroergotamine, the analysis of the interaction profile over the 60 ns trajectory shows that five residues are the most common, Glu466 (HB: 99.34%), Ile339 (HI: 94.52%), Val59 (HI: 94.02%), Ile107 (HI: 81.56%), and His461 (HB: 58.64%). For the short stable lapse (72–100 ns), flucytosine maintained its profile that was observed to occur with Asn340 (HB: 90.75%), Tyr455 (HB: 59.43%), Pro336 (HI: 58.72%), and Arg472 (HB: 55.52%).

#### 2.5.2. Root Mean Square Fluctuation (RMSF) Analysis

**Figure 5:**
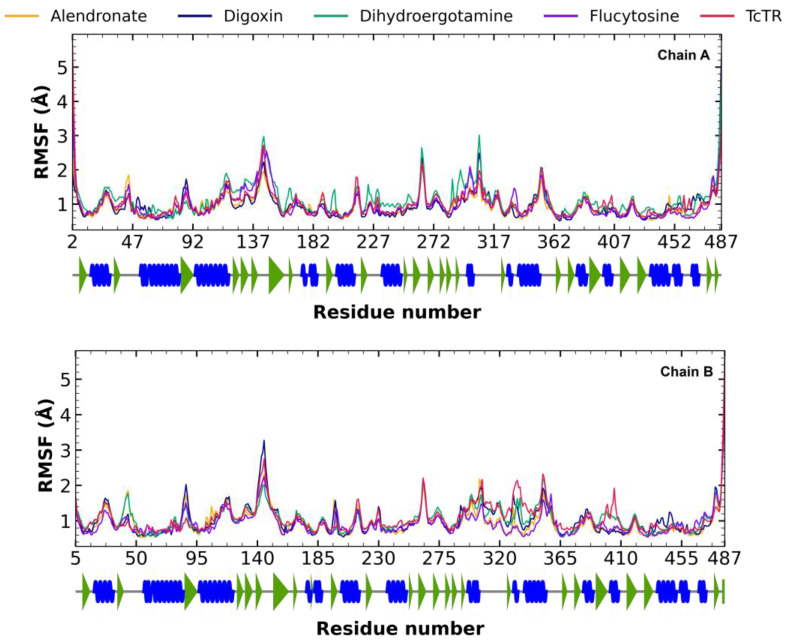
The RMSF graph for fluctuations over time for *Tc*TR complex fluctuation: alendronate, digoxin, dihydroergotamine, flucytosine, and apo-*Tc*TR. Blue spiral (alpha helix), green triangle (beta sheet), in between space (loop).

The RMSF fluctuations for ligand–*Tc*TR complexes were determined; the alendronate–*Tc*TR complex (orange), digoxin–*Tc*TR (blue), dihydroergotamine–*Tc*TR (green), flucytosine–*Tc*TR (violet), and free *Tc*TR (red) are shown in Figure 5. RMSF graphs for TR show minor fluctuations located at loop regions, which are prone to fluctuations; still, proteins remain mostly stable through the dynamics, suggesting that ligand interaction did not considerably affect proteins. Altogether, this RMSF supports the notion that the complexes formed with each ligand are stable at the site each binds to and do not significantly alter the residue fluctuations at any regions.

#### 2.5.3. Radius of Gyration Analysis

**Figure 6:**
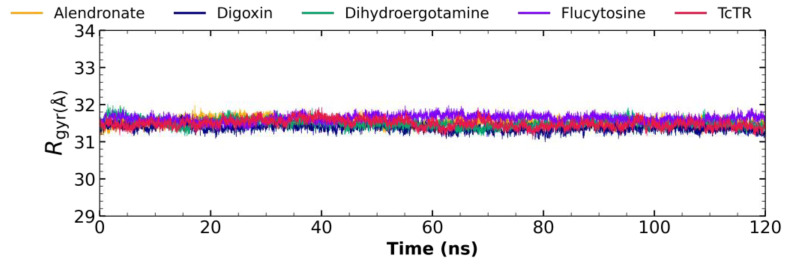
The radius of the gyration graph for molecular dynamics over time for alendronate (orange), digoxin (blue), dihydroergotamine (green), flucytosine (violet), and apo-*Tc*TR (red).

Figure 6 shows the radius of gyration for ligand–*Tc*TR complexes: the alendronate–*Tc*TR complex (orange), digoxin–*Tc*TR (blue), dihydroergotamine–*Tc*TR (green), flucytosine–*Tc*TR (violet), and apo-*Tc*TR. Complexes remain stable throughout the 120 ns of molecular dynamics with minimal fluctuation; comparing the receptor to the complexes, there is not a major difference, which suggests that the protein remains compact in its dynamics.

### 2.6. In Vitro Activity on Blood Trypomastigotes

**Figure 7:**
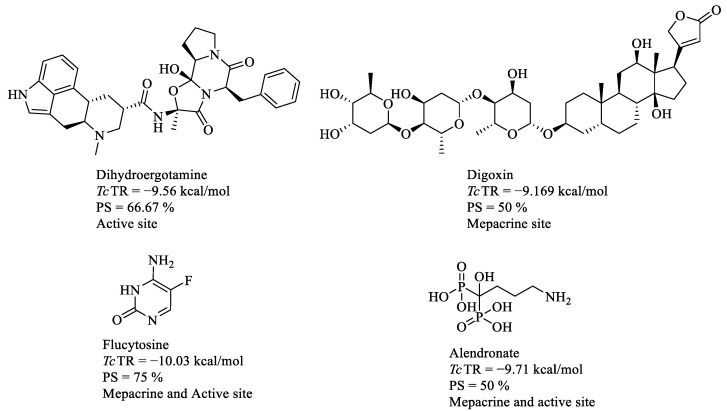
Four selected FDA-approved drugs for biological evaluation against trypomastigotes of *T. cruzi* in an in vitro model.

Four drugs (Figure 7) were selected and evaluated at a single concentration to find those with a lysis percentage equal to or higher than the reference drugs on trypomastigotes present in the blood of infected mice (Table 2). Flucytosine has the lowest percentage of lysis on the NINOA strain and alendronate on the INC-5 strain. However, they present similar trypanocidal values to the reference drugs against the NINOA strain; therefore, the half-maximal lytic concentration (LC_50_) of all drugs was determined in both strains. Alendronate, digoxin, and dihydroergotamine presented LC_50_ values similar or better than the reference drugs.

### 2.7. Short-Term In Vivo Assay in a Murine Model of T. cruzi Infection

**Figure 8:**
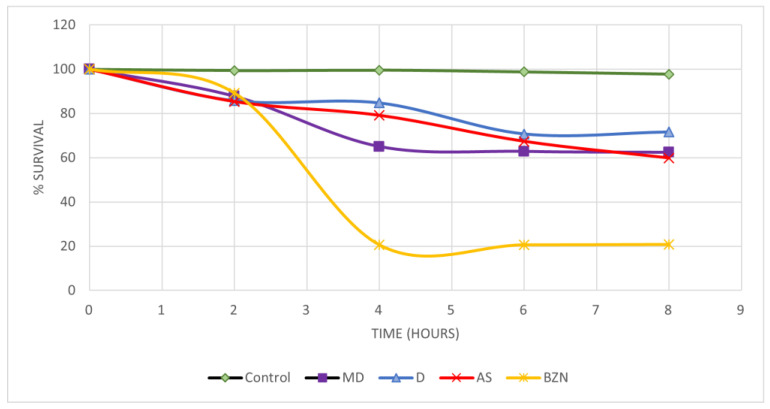
The percentage of parasite survival induced by three FDA drugs and benznidazole treatments in a short-term in vivo model of trypanosomiasis (NINOA strain). MD: Dihydroergotamine, D: digoxin, AS: alendronate. BZN reference drug. The control is infection without treatment.

The results obtained from the evaluation of alendronate, digoxin, and dihydroergotamine mesylate in a short-term in vivo model of infection with the *T. cruzi* NINOA strain are shown in Figure 8

## 3. Discussion

In general, in the molecular docking analysis on three active sites of *Tc*TR (Table 1), some drugs appear as potential ligands in two out of the three docked sites, and four drugs are predicted with a high inhibitory potential on the three active sites: digoxin, flucytosine, regorafenib, and vilazodone. These results could be an advantage since in this way, the enzyme could simultaneously be inhibited at different sites, resulting in a better inhibition of the enzyme, leaving the parasite more vulnerable to ROS. However, only two drugs were selected, digoxin and flucytosine, due to reasons that are mentioned specifically for each site.

### 3.1. Molecular Docking on Z Site

The interaction profiles observed for the binding site of the control compounds were used for comparison with the FDA-approved drugs to test their potential to binding *Tc*TR. The most frequently occurring amino acids interactions were eight (HI: Val195, Phe199, Tyr222, Arg223, and Ile286. HB: Gly197, Arg223, and Arg229). Ten FDA-approved drugs (digoxin, tannic acid, paclitaxel, dibucaine, nafarelin, conivaptan, flucytosine, edrophonium, tolvaptan, and ganirelix) had the best docking score value with a percentage of similarity (≥40%) of the ligand–protein interaction profile, which is presented at the right edge of Figure 1, where the percentage of similarity between the ligands is presented in comparison with the amino acids mentioned above (those that occur most frequently for control compounds in this site); summary interaction profiles may be consulted in the Supplementary Materials. However, some drugs have a molecular weight (>1000 g/mol) and cause several adverse effects or could be potential pan-assay interference compounds (PAINS), which tend to interact nonspecifically with many biological targets, generating false positives; such criteria are described in Supplementary Table S1. Therefore, only digoxin (Figure 4) was proposed for further in vitro studies. Digoxin is a digitalis drug used to treat arrhythmias; there are no reports of having been tested on *T. cruzi*.

### 3.2. Molecular Docking on Mepacrine Site

An analysis of the ligand–protein interactions of the top 20 FDA-approved drugs was carried out, and a comparison was made with the set of ligand–protein interaction profiles of the controls used for this site (Figure 2). Five amino acid residues were the most frequently shared (HI: Leu18, Trp22, Ile339, and Leu399 and HB: Tyr 111), Try111, Ile339, and Leu399 being the most recurrent. The FDA-approved drugs with the best docking score value and PS ≥ 40% were posaconazole, digitoxin, flucytosine, alendronate, digoxin, cetrorelix, vilazodone, metocurine, anidulafungine, and plerixafor. However, from the previous criteria applied (Supplementary Table S2), only flucytosine and alendronate (Figure 4) were selected for further studies. Alendronate is used to treat osteoporosis, and it has been previously tested as an inhibitor of *T. cruzi* farnesyl diphosphate synthase [11,12], whereas flucytosine, an antifungal drug, has also not been tested against *T. cruzi*.

### 3.3. Molecular Docking on the Catalytic Site

Out of the top 20 FDA-approved drugs considering the percentage similarity of the ligand–protein interaction profile with the controls was performed, and the amino acid residues with the highest occurrence are six in number (HI: Leu18, Ile107, Ile339, Leu399, and Pro462 and HB: Glu466). The amino acid residues with the highest frequency were Ile339, Phe396, Pro398, Leu399, and Pro462. The drugs with the best binding score value and PS ≥ 40% were flucytosine, digitoxin, telmisartan, nilotinib, zafirlukast, alendronate, digoxin, and dihydroergotamine (Supplementary Table S3), although only dihydroergotamine (Figure 4) was selected for in vitro studies. Dihydroergotamine is used to treat migraines, and it has not been reported to be tested against *T. cruzi*.

### 3.4. Molecular Dynamic Analysis

The behavior for the complexes for alendronate and digoxin with *Tc*TR suggests that these have the highest potential to behave as *Tc*TR inhibitors, as throughout the full 120 ns trajectory, these show only minor fluctuations regarding their RMSD, under 2.5 Å. Considering that the interactions for alendronate, as both Val54 and Val59, which have a frequency over 70%, are neighboring to catalytic Cys53 and Cys58 and, additionally, His461, the remaining catalytic triad residue, participates in nearly 60% of the analyzed frames, it suggests that alendronate, while it maintains a significantly stable pose, could potentially block the access of trypanothione disulfide to the catalytic triad. The most frequent interactions for digoxin occur with residues reported to interact with NADPH (Gly197, Gly198, Phe199, Arg229, Ile286); thus, digoxin may act as a molecule that limits the access for the electron carrier and may inhibit the reductase activity. Second, the behavior observed for dihydroergotamine shows a mild potential to act as a *Tc*TR inhibitor, as though it shows instability initially, after the first third (40 ns) of the dynamic trajectory, it attains a relative stability at about 4.5 Å, which is maintained to the end of the trajectory. Dihydroergotamine maintains the interaction with Val59 over 90% of the frames analyzed; as this is a catalytic neighboring residue, it may be suggested that it could potentially hinder the access of trypanothione disulfide to the catalytic residues. Additionally, dihydroergotamine bears an interaction with His461 (a catalytic residue) for nearly 60% of the analyzed frames, altogether suggesting that the pose it attains after fluctuating during the first 40 ns is an orientation that may result in being detrimental to the reductase activity. Flucytosine shows the least stability for the ligand–*Tc*TR complex, setting it as the least probable candidate to act as a *Tc*TR inhibitor; thus, it is likely to act using a different mechanism. For flucytosine, the interacting residues reside at the cavity entrance, thus potentially blocking the access to the trypanothione disulfide, yet the stability is low; thus, its potential to act as a *Tc*TR inhibitor is low. Both the analysis of RMSF and the radius of gyration show that these molecules do not significantly alter either the residue fluctuations at their respective docking sites nor do they destabilize the protein’s 3D conformation, as its radius of gyration remains compact throughout. Altogether, this analysis supports the initial proposal (proposed using molecular docking protocol) of alendronate, digoxin, and dihydroergotamine as potential *Tc*TR inhibitors.

### 3.5. In Vitro Activity on Blood Trypomastigotes

The results regarding screening (Table 1) show that all the drugs have a lysis percentage comparable to the reference drugs, indicating that their trypanocidal activity is present; for this reason, a dose-dependent trial was continued using different concentrations to determine the LC_50_. As shown in Table 2 flucytosine had an LC_50_ above the reference drugs; for this reason, it was discarded to continue its evaluation in vivo, while the remaining three drugs show results that warrant further studies. These results become relevant since blood trypomastigotes is the morphology present in the mammalian host and thus responsible for the acute form of Chagas disease.

### 3.6. Short-Term In Vivo Assay in a Murine Model of T. cruzi Infection

The control Bzn was the drug with the lowest blood parasite survival after 8 h drug administration (20.83%), followed by alendronate with 59.83%, dihydroergotamine mesylate with 62.44%, and finally digoxin with 71.60% parasite survival. The Kruskal–Wallis statistical test determined that there is a significant difference between the three drugs tested and the positive control, Bzn (*p* < 0.01); however, when comparing the FDA-approved drugs, we can observe that there is no significant difference between them (*p* > 0.05). It is observed that the percentage of parasite clearance by the drugs is lower than the control drug; this could be due to the requirements of the metabolism and absorption of the drugs. Notwithstanding, they did present an effect on parasitemia, suggesting that they can be evaluated in combined therapies with Bzn to determine a possible synergistic effect to reduce the treatment time or the dose of the Bzn to achieve a reduction in the adverse effects.

## 4. Materials and Methods

### 4.1. Protein Preparation

The three-dimensional structure of *Tc*TR was obtained from the Protein Data Bank (PDB) database with the accession code 1BZL [13]. The protein was prepared in the open access software UCSF-Chimera 1.16 [14], with which all co-crystallized molecules were removed; subsequently, hydrogens and charges were added using the UCSF-Chimera built-in Dock Prep tool and finally converted to PDBQT format by adding the Gasteiger charges with the MGTools 1.5.7 tool. The force field applied was AMBER [15].

### 4.2. Ligand Library Preparation

The FDA drugs were obtained from the BindingDB database [16] in a single file in .tsv format, then using the Open Babel 3.1 [17] software, all those repeated were eliminated, and with the same tool, the file was converted to .smi format. Then, each drug was separated into individual files and converted to .sdf format; then, the molecules were minimized and converted to .mol2 format, and the force field was MMFF94 [15].

### 4.3. Molecular Docking

The software used to perform molecular docking of all ligands was AutoDock Vina 1.1.2 [18]. Molecular docking was performed on three distinct active sites: catalytic site, with coordinates X = 24.663, Z = 9.125, and Y = −4.301, obtained through selection of the center of mass of one of the two trypanothione molecules present in 1BZL with PyMOL 3.0.3 [19]; mepacrine site, with coordinates X = 17.733, Z = 13.499, and Y = −1.197; and the site centered at the Z site, with coordinates X = 41.825, Y = 4.351, and Z = −28.343, reported previously (Espinosa-Bustos et al., 2022) [20]. The box size was calculated for each of the different ligands according to the radius of gyration of each ligand (Feinstein & Brylinski, 2015) [21].

### 4.4. Molecular Docking Analysis

A series of controls (Biscriptolepine, Komaroviquinone, ZINC12151998, 3-Methoxycarpachromene, JWZ, M9Y, M9J, RD0, RD7, M9S, WPE, JV0, WP7, 2JR, WP6, QUM, WP5, WPF, TS8, RDS, diverse quinone derivatives) (Battista et al., 2020; Espinosa-Bustos et al., 2022; Maamri et al., 2021; Matadamas-Martínez et al., 2019; Saha & Sharma, 2015) [9,20,22,23,24] were gathered from different literature reports of *Tc*TR inhibitors and were docked to *Tc*TR at the three docking sites of interest. The docking score threshold values and the most common interaction profiles were obtained from this initial analysis this interaction profile was used as consensus profile to contrast with the profiles for the FDA-approved ligands; full profiles may be consulted in Supplementary Materials. Further on, molecular docking was analyzed using two criteria: binding energy and protein–ligand interaction profile. The binding energy of the ligands was compared with the controls (Supplementary Materials) that were downloaded from the different databases; from this comparison, the drugs that were either above or very close to the binding energy of the controls were selected, and the top ten drugs were selected from each of the sites mentioned above. For the interaction profile, the comparison was made against the controls (Supplementary Materials), selecting those amino acid residues that appear more frequently among the interactions of the drugs and controls with the proteins presented at the right edge of Figure 1–3 column as percentage of similarity (PS); likewise, those FDA drugs that shared these amino acids in greater proportion were selected.

### 4.5. Molecular Dynamic Analysis

Once the four drugs were chosen, molecular dynamics was carried out in GROMACS software version 2018 using the AMBER03 force field. The topology of each of the chosen drugs was generated with the ACPYPE 2022.1.3 antechamber module with the General Amber Force Field. Solvation was carried out with water molecules in a dodecahedron with the minimum distance from the wall of 10 Å, and the TIP3P water model was used. Next, chlorine and sodium ions were added to neutralize the system with a 50,000-fold energy minimization. Two equilibrium steps were then carried out at 300 K. In the first, the drug was simulated at NVT conditions (constant number of particles, volume, and temperature). For the second step, the drug was simulated at NTP conditions (constant number of particles, pressure, and temperature). Each step reaches duration of 100 ps. The simulation was performed at a temperature of 300 K for a 120 ns trajectory using a V-rescale thermostat and a Parrinello–Rahman barostat with tau_ and tau_p, respectively. The calculation for long-range electrostatic interactions was performed using the Particle Mesh Ewald method, and the LINCS algorithm was used to know the H-bond length restrictions. To determine the stability of each complex, the root mean square deviation (RMSD), the root mean square fluctuation (RMSF), and the radius of gyration (gyr) were obtained (González-González et al., 2023) [25].

### 4.6. In Vitro Trypanocidal Assay of Blood Trypomastigotes

Parasite obtention was performed using female mice of the CD1 strain weighing 25–35 g, which were infected with blood trypomastigotes intraperitoneally, one group with the NINOA strain and the other with the INC-5 strain of *T. cruzi*. Once the maximum peak of parasitemia was reached (approximately 2–4 weeks), infected blood was extracted by cardiac puncture using sodium heparin as anticoagulant, and this blood was adjusted to 1 × 10^6^ trypomastigotes/mL (diluted with saline) (Chacón-Vargas et al., 2017) [26]. First, a screening was performed to select drugs with trypanocidal activity; in a 96-well microplate, 90 µL of the previously adjusted blood and 10 µL of the selected FDA drugs were added at a final concentration of 12.5 µg/mL. In parallel, the reference drugs, Nfx and Bzn, were used as positive controls at the same concentration (12.5 µg/mL); same volume of the vehicle in which the drugs were dissolved (DMSO 1%) was used as negative control. Each drug and reference were mounted in triplicate. The microplate was incubated at 4 °C for 24 h; then, the trypomastigote count was performed using the Pizzi–Brener method, which consists of taking 5 µL of blood from the wells, putting it on a slide, and covering it with an 18 × 18 mm coverslip; 15 random fields were counted under a brightfield optical microscope at a magnification of 40×. The percentage of lysis by each compound was obtained by comparing them with the live trypomastigotes of the negative control (no drug) (Juarez-Saldivar et al., 2024) [27]. Those compounds whose percentage of lysis was close or higher than the reference drugs (Nfx and Bzn) were selected to continue with the tests and calculation of the half-maximal lytic concentration (LC_50_). For this, the compounds were tested at five concentrations in serial dilutions (100–6.25 µg/mL); the percentage of lysis was determined, and the computation of the LC_50_ was determined using the Probit tool. Later, the results were converted to micromolar units [28].

### 4.7. Short-Term In Vivo Assay

For the in vivo assay, mice of the CD1 strain weighing 30–40 g were inoculated intraperitoneally with blood trypomastigotes at a concentration of 1 × 10^5^ trypomastigotes/mL, with modifications to the methodology reported by Romanha et al. [29] and Wong-Baeza et al. [30], one group with the NINOA strain and another group with the INC-5 strain. After the necessary days to reach the necessary parasitemia (12–18 dpi), they were separated into 5 groups, each consisting of three mice; the first group was administered with only 4% gum Arabic (vehicle medium for the reference drugs and medicines), the second group Bzn, the third group dihydroergotamine, the fourth group digoxin, and the fifth group alendronate. The dose administered was 100 mg/kg. From this moment on, counted as zero hour, 5 μL of blood was taken from each mouse from the caudal vein; the trypomastigotes count was performed as described above, and the procedure was repeated at two, four, six, and eight hours. The percentage of lysis of each compound was calculated from the comparison of the number of blood trypomastigotes counted compared to time zero for each of the groups.

## 5. Conclusions

This study, based on a molecular docking approach to evaluate a library of FDA-approved drugs at three different sites of the pharmacological target *Tc*TR, allowed us to identify alendronate, digoxin, and dihydroergotamine as potential anti-*T. cruzi* drugs by presenting similar or better LC_50_ values than the reference drugs in an in vitro model; although it is observed that these drugs have this activity, it is necessary to demonstrate in future studies through enzymatic assays that it is acting on the *Tc*TR enzyme or if these may act through an alternative mechanism. However, the effect was not pronounced in the short-term in vivo assay in an animal model; although they did show a trypanocidal effect, it did not reach that of the canonical antichagasic, Bzn. This opens the possibility of combined therapies to reduce the adverse effects of Bzn.

## Figures and Tables

**Table 1:** The docking score (DS) of the top 20 FDA-approved drugs and reference inhibitors on the three molecular docking sites.

Z Site		Mepacrine Site		Catalytic Site	
Drug	DS (kcal/mol)	Drug	DS (kcal/mol)	Drug	DS (kcal/mol)
Digoxin	−10.148	Dactinomycin	−11.136	Flucytosine	−11.725
Anidulafungine	−9.873	Irinotecan	−10.64	Digitoxin	−11.012
Tannic acid	−9.551	Posaconazole	−10.381	Dactinomycin	−10.973
Metocurine	−9.238	Digitoxin	−10.379	Irinotecan	−10.489
Nilotinib	−9.203	Tannic acid	−10.075	Anidulafungin	−10.44
Palperidone	−9.186	Flucytosine	−10.034	Telmisartan	−10.334
Crizotinib	−9.169	Regorafenib	−9.731	Nilotinib	−10.264
Paclitaxel	−9.158	Tetracaine	−9.58	Regorafenib	−9.899
Dibucaine	−9.085	Alendronate	−9.437	Vilazodone	−9.849
Nafarelin	−9.051	Sorafenib	−9.34	Dutasteride	−9.832
Conivaptan	−9.049	Telbivudine	−9.303	Lanreotide	−9.802
Ponatinib	−9.047	Digoxin	−9.169	Zafirlukast	−9.777
Flucytosine	−9.031	Dutasteride	−9.156	Lapatinib	−9.77
Lomitapide	−8.979	Temsirolimus	−9.082	Sorafenib	−9.748
Edrophonium	−8.969	Cetrorelix	−9.009	Tetracaine	−9.718
Itraconazole	−8.945	Lanreotide	−8.936	Alendronate	−9.714
Vilazodone	−8.881	Vilazodone	−8.817	Ponatinib	−9.627
Regorafenib	−8.878	Metocurine	−8.801	Telbivudine	−9.569
Tolvaptan	−8.866	Anidulafungine	−8.758	Digoxin	−9.55
Ganirelix	−8.864	Plerixafor	−8.717	Dihidroergotamine	−9.536
**ZINC12151998**	**−10.3**	**JWZ**	**−8.914**	**ZINC12151998**	**−10.799**
**7i**	**−9.838**				
**7e**	**−9.428**				

**Table 2:** The percentage of lysis and LC_50_ values of four FDA drugs on *Trypanosoma cruzi* strains NINOA and INC-5.

Drug	% Lysis at 12.5 µg/mL		LC50 µmol	
	NINOA	INC-5	NINOA	INC-5
Flucytosine	13.4 ± 2.9	21.9 ± 1.5	613 ± 22	1272 ± 59
Alendronate	28.2 ± 6	17.3 ± 15.3	174 ± 11	277 ± 13.3
Digoxin	36.2 ± 2	33.6 ± 2.5	45 ± 2.8	76 ± 10
Dihydroergotamine	29.4 ± 1.5	25 ± 7	28.1 ± 3.1	57 ± 2.6
Nifurtimox	28.6 ± 4.5	25 ± 3.6	161 ± 33	255 ± 39
Benznidazole	32.2 ± 6	30.3 ± 5.5	220 ± 40	337 ± 34

## Data Availability

Data are contained within the article.

## Supplementary Materials

The following supporting information can be downloaded at https://www.mdpi.com/article/10.3390/molecules29163796/s1, Tables S1–S3, Controls used for TR, and Interaction profiles for docked control *Tc*TR inhibitors.
