# Supplementary material for: Molecular Docking-Based Virtual Screening of FDA-Approved Drugs Using Trypanothione Reductase Identified New Trypanocidal Agents
Source: Molecules. 2024 Aug 10;29(16):3796. doi: 10.3390/molecules29163796 (PMC11357579; doi:10.3390/molecules29163796)

Table S1. Reasons for ruling out Z site drugs of *Tc*TR.

DRUG	REASONS TO DISCARD
**DIGOXIN**	N/A
**ANIDULAFUNGIN**	- Molecular weight greater than 1000 g/mol, considered as PAINS<br>-High Costs<br>- Need for specialized personnel for application
**TANNIC ACID**	- Molecular weight greater than 1000 g/mol, considered as PAINS
**METOCURINE**	- Similarity percentage less than 40%<br>- Molecular weight greater than 1000 g/mol, considered as PAINS<br>- Need for specialized personnel for application<br>- It has fallen into disuse because there are new, safer alternatives.
**NILOTINIB**	- Similarity percentage less than 40%<br>- High Price<br>- The side effects they present are worse than the reference drugs
**PALIPERIDONE**	- Similarity percentage less than 40%<br>-High Price<br>- The side effects they present are worse than the reference drugs
**CRIZOTINIB**	- Similarity percentage less than 40%<br>-High Price<br>- The side effects they present are worse than the reference drugs
**PACLITAXEL**	- The side effects they present are worse than the reference drugs<br>- High price
**DIBUCAINE**	- Topical administration, which makes contact with the parasite difficult.
**NAFARELINE**	- Molecular weight greater than 1000 g/mol, considered as PAINS<br>- High Price
**CONIVAPTAN**	- Need for specialized personnel for application<br>- High Price
**PONATINIB**	- Similarity percentage less than 40%<br>- High Price<br>- The side effects they present are worse than the reference drugs
**FLUCYTOSINE**	- Has already been selected on the mepacrine site
**LOMITAPIDE**	- Similarity percentage less than 40%<br>-High Price<br>- The side effects they present are worse than the reference drugs
**EDOPHONIUM**	- The side effects they present are worse than the reference drugs
**ITRACONAZOLE**	- Similarity percentage less than 40%
**VILAZODONE**	- Similarity percentage less than 40%
**REGORAFENIB**	- Similarity percentage less than 40%<br>- High hepatotoxicity
**TOLVAPTAN**	- May cause dehydration<br>- requires constant medical supervision
**GANIRELIX**	- Molecular weight greater than 1000 g/mol, considered as PAINS<br>- High Price

Table S2. Reasons for ruling out mepacrine site drugs of *Tc*TR

DRUG	REASONS TO DISCARD
**DACTNOMYCIN**	- Similarity percentage less than 40%<br>- Molecular weight greater than 1000 g/mol, considered as PAINS<br>-High Costs<br>- Need for specialized personnel for application
**IRINOTECAN**	- Similarity percentage less than 40%<br>- The side effects they present are worse than the reference drugs<br>- Need for specialized personnel for application<br>-High Cost
**POSACONAZOLE**	- It has been tested several times before
**DIGITOXIN**	- Has fallen into disuse, replaced by digoxin, making it difficult to obtain
**TANNIC ACID**	- Similarity percentage less than 40%<br>- Molecular weight greater than 1000 g/mol, considered as PAINS
**FLUCYTOSINE**	N/A
**REGORAFENIB**	- Similarity percentage less than 40%<br>- High hepatotoxicity
**TETRACAINE**	- Similarity percentage less than 40%<br>- Due to its form of application (topical), it is unlikely to have any effect or contact with blood trypomastigotes.
**ALENDRONATE**	N/A
**SORAFENIB**	- Similarity percentage less than 40%<br>- The side effects they present are worse than the reference drugs<br>- High Price
**TELBIVUDINE**	- Similarity percentage less than 40%<br>- High hepatotoxicity<br>- High Price
**DIGOXIN**	- Previously selected for site Z
**DUTASTERIDE**	- Similarity percentage less than 40%
**TEMSIROLIMUS**	- Similarity percentage less than 40%<br>- Molecular weight greater than 1000 g/mol, considered as PAINS<br>-High Costs<br>- Need for specialized personnel for application<br>- The side effects they present are worse than the reference drugs
**CETRORELIX**	- Similarity percentage less than 40%<br>- Molecular weight greater than 1000 g/mol, considered as PAINS<br>- The side effects they present are worse than the reference drugs<br>- High hepatotoxicity
**LANREOTIDE**	- Similarity percentage less than 40%<br>- Molecular weight greater than 1000 g/mol, considered as PAINS<br>- High Cost<br>-20-33% can develop cholelithiasis
**VILAZODONE**	- Its use in children under 18 years of age is not recommended, because in some cases, the appearance of suicidal tendencies has been reported<br>- In Mexico and Latin America it is not easy to obtain for those people who could use it as a treatment.

	- Requires medical follow-up, which given the circumstances in rural areas (where most cases occur) it would be very difficult to comply.
**METOCURINE**	- Similarity percentage less than 40%<br>- Molecular weight greater than 1000 g/mol, considered as PAINS<br>- Need for specialized personnel for application<br>- It has fallen into disuse because there are new, safer alternatives.
**ANIDULAFUNGINE**	- Molecular weight greater than 1000 g/mol, considered as PAINS<br>-High Costs<br>- Need for specialized personnel for application
**PLERIXAFOR**	-High Costs<br>- Need for specialized personnel for application

Table S3. Reasons for ruling out catalytic site drugs of *Tc*TR

DRUG	REASONS TO DISCARD
**FUCYTOSINE**	- Has already been selected on the mepacrine site
**DIGITOXIN**	- Has fallen into disuse, replaced by digoxin, making it difficult to obtain
**DACTINOMICYN**	- Similarity percentage less than 40%<br>- Molecular weight greater than 1000 g/mol, considered as PAINS<br>-High Costs<br>- Need for specialized personnel for application
**IRINOTECAN**	- Similarity percentage less than 40%<br>- The side effects they present are worse than the reference drugs<br>- Need for specialized personnel for application<br>-High Cost
**ANIDULAFUNGIN**	- Molecular weight greater than 1000 g/mol, considered as PAINS<br>-High Costs<br>- Need for specialized personnel for application
**TELMISARTAN**	- Safety profiles have not been conducted for all age groups.
**NILOTINIB**	- High Price<br>- The side effects they present are worse than the reference drugs
**REGORAFENIB**	- Similarity percentage less than 40%<br>- High hepatotoxicity
**VILAZODONE**	- Similarity percentage less than 40%
**DUTASTERIDE**	- Similarity percentage less than 40%
**LANREOTIDE**	- Similarity percentage less than 40%<br>- Molecular weight greater than 1000 g/mol, considered as PAINS<br>- High Cost<br>-20-33% can develop cholelithiasis
**ZAFIRLUKAST**	- Adverse effects similar to reference drugs
**LAPATINIB**	- Similarity percentage less than 40%<br>- High Cost<br>-Adverse effects similar to reference drugs
**SORAFENIB**	- Similarity percentage less than 40%<br>- The side effects they present are worse than the reference drugs<br>- High Price
**TERTRACAINE**	- Similarity percentage less than 40%<br>- Due to its form of application (topical), it is unlikely to have any effect or contact with blood trypomastigotes.
**ALENDRONATE**	- Has already been selected on the mepacrine site
**PONATINIB**	- Similarity percentage less than 40%<br>-High Price<br>- The side effects they present are worse than the reference drugs
**TELBIVUDINE**	- Similarity percentage less than 40%<br>- High hepatotoxicity<br>- High Price
**DIGOXIN**	- Previously selected for site Z
**DIHYDROERGOTAMINE**	N/A

Controls used for TR

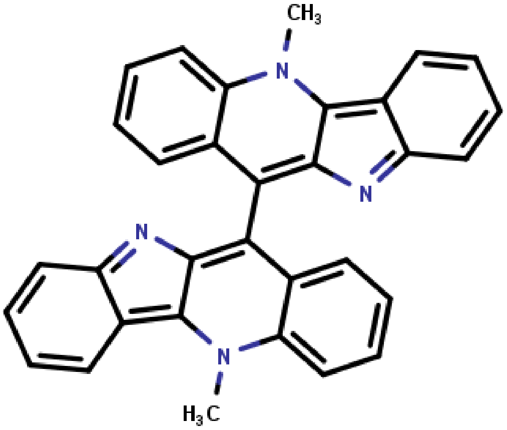

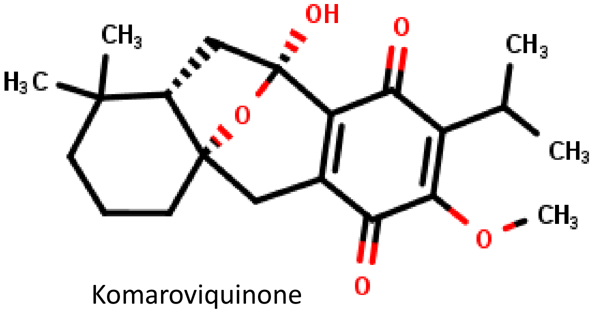

Komaroviquinone
IC_{50} = 1 µg/ml

Biscriptolepine
IC_{50} = 3.48 µg/ml

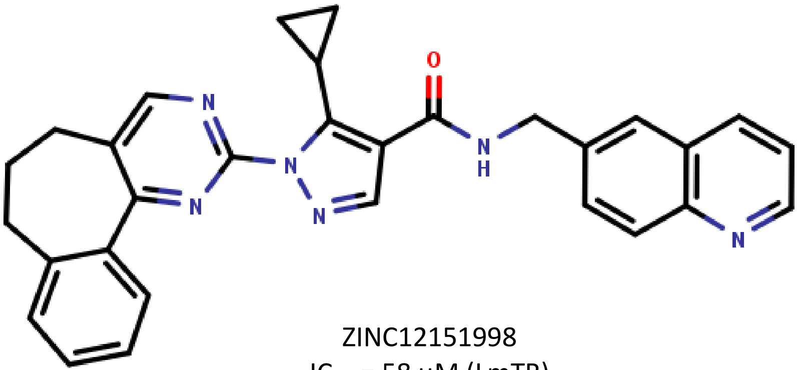

ZINC12151998
IC_{50} = 58 µM (LmTR)
CC_{50} = 53 µM

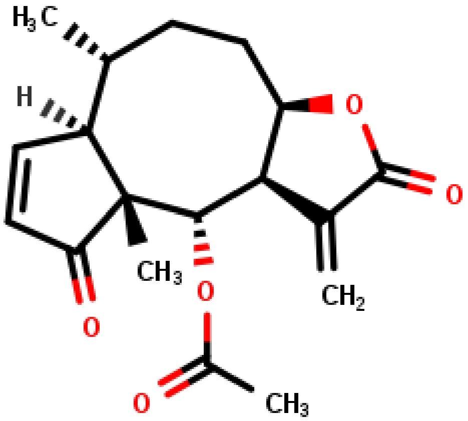

(1R,2S,3R,7R,10R,11R)-1,10-dimethyl-4-methylidene-5,14-dioxo-6-oxatricyclo[9.3.0.0^{3,7}]tetradec-12-en-2-yl acetate
IC_{50} = 0.54 µM *(T. cruzi)*
IC_{50} = 0.063 (*T.brucei rhodesiense)* µM

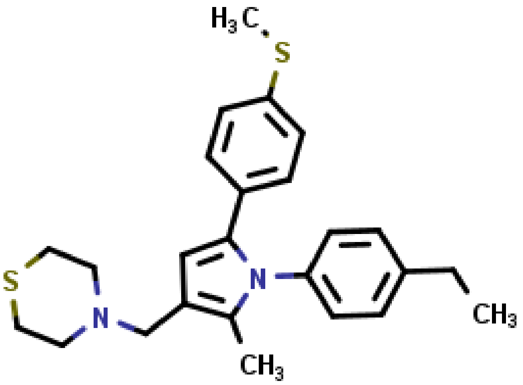

JV0
IC_{50} = 13.8 µM (Li)

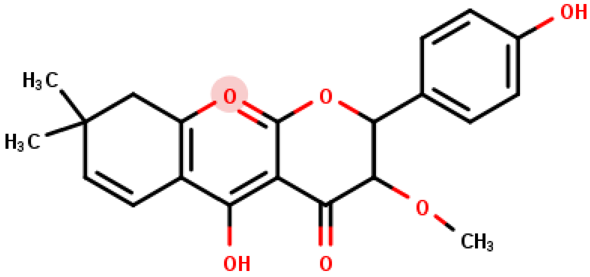

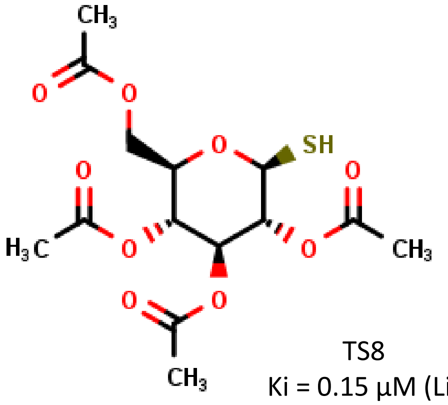

TS8
Ki = 0.15 μM (Li)

3-Methoxycarpachromene
Ki (teórico) = 0.038 M (LiTR)

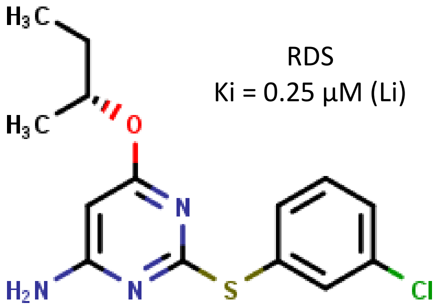

RDS
Ki = 0.25 μM (Li)

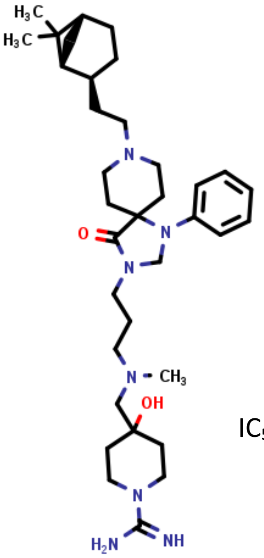

JWZ
IC_{50} = 5.7 μM

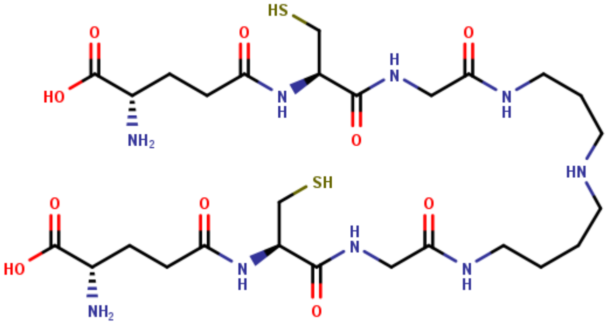

Trypanothione
Ki = 0.25 μM (Li)

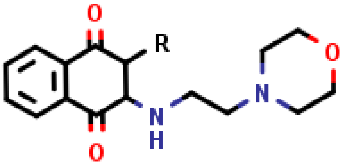

Quinone derivatives
2a IC_{50} = 3.06 μM. R=H
2b IC_{50} = 2.98 μM. R=Cl
2c IC_{50} = 2.22 μM. R=Br

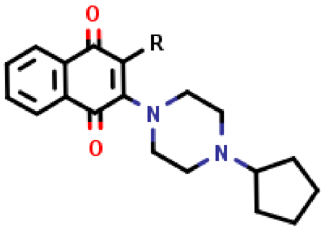

Quinone derivatives
2d IC_{50} = 5.04 μM. R=H
2e IC_{50} = 0.19 μM. R=Cl
2f IC_{50} = 1.46 μM. R=Br

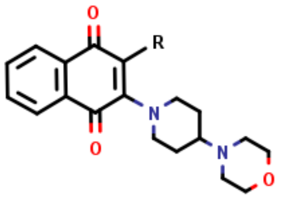

Quinone derivatives
2g IC$_{50}$ = 8.45 µM. R=H
2h IC$_{50}$ = 6.60 µM. R=Cl
2i IC$_{50}$ = 1.74 µM. R=Br

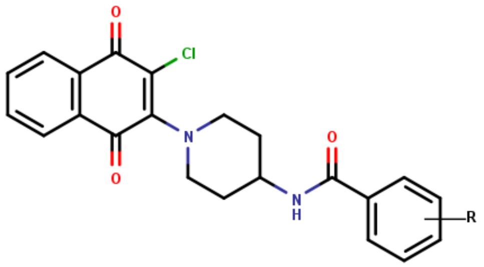

Quinone derivatives
7a IC$_{50}$ = 2.46 µM. R=2-Br
7b IC$_{50}$ = 2.23 µM. R=4-NO_2
7c IC$_{50}$ = 1.14 µM. R=3-Cl
7d IC$_{50}$ = 2.30 µM. R=4-CH_2Cl
7e IC$_{50}$ = 5.6 µM. R=3,5-CH_3
7f IC$_{50}$ = 1.84 µM. R=3,5-NO_2
7g IC$_{50}$ = 2.57 µM. R=3-Cl,5-F
7h IC$_{50}$ = 1.5 µM. R=3-F,4-OCH_3
7i IC$_{50}$ = 3.35 µM. R=2-NO_2,4-CF_3

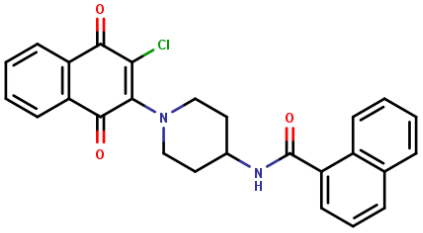

7j
IC$_{50}$ = 0.92 µM

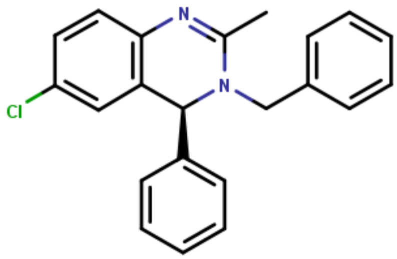

WP6
IC$_{50}$ = 0.93 µM

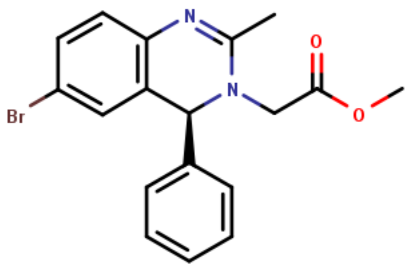

WP5
IC$_{50}$ = 6.8 µM

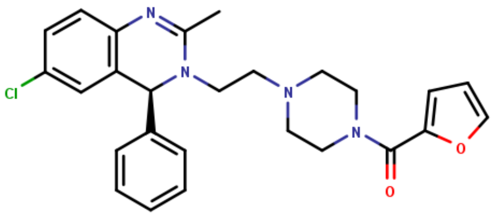

WP7
IC$_{50}$ = 0.42 µM

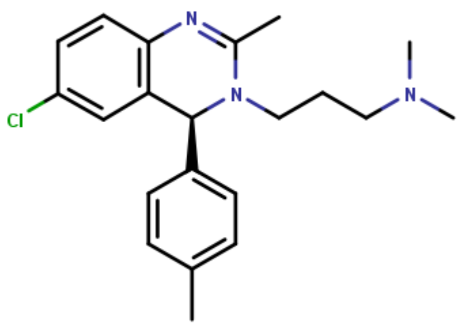

WPF
IC$_{50}$ = 0.23 µM

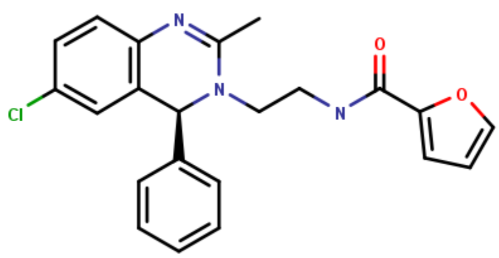

WPE
IC$_{50}$ = 0.86 µM

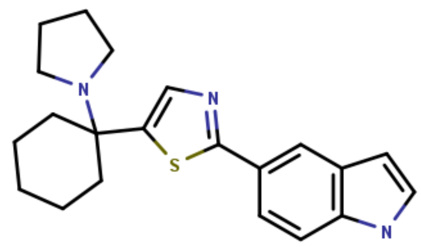

2JR
% Inhibition = 79% (*Tc*TR)

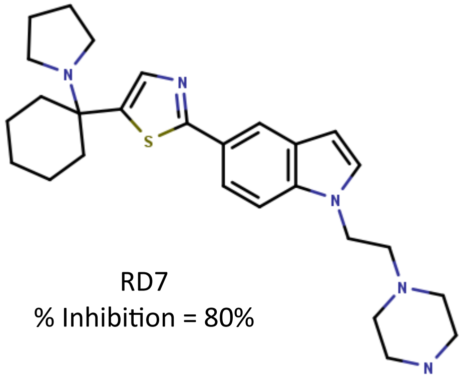

RD7
% Inhibition = 80%

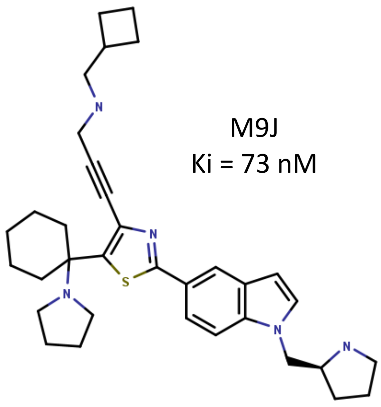

M9J
Ki = 73 nM

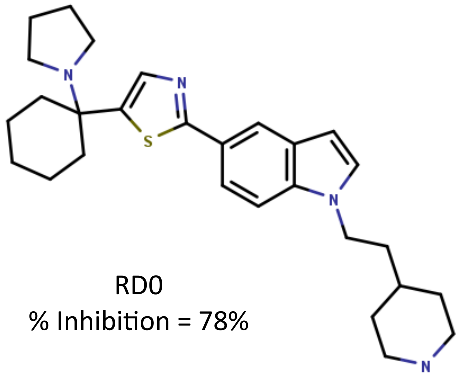

RD0
% Inhibition = 78%

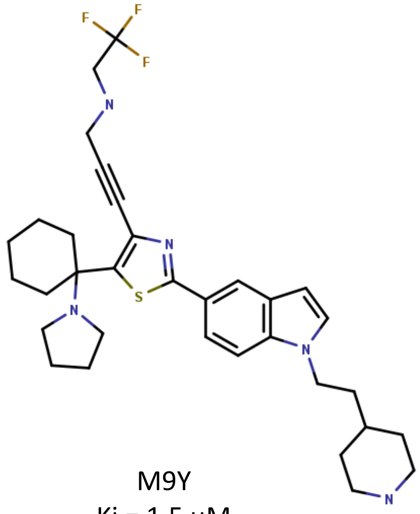

M9Y
Ki = 1.5 µM

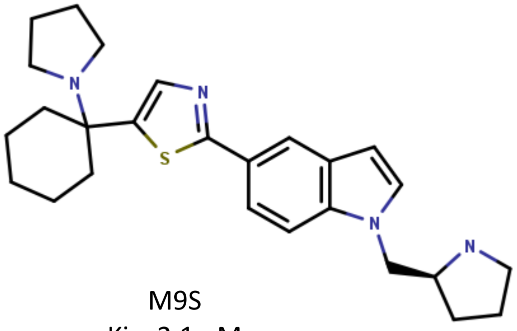

M9S
Ki = 2.1 µM

Interaction profiles for docked control *Tc*TR inhibitors

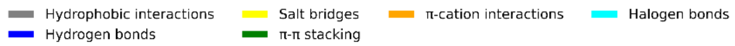

Z-site

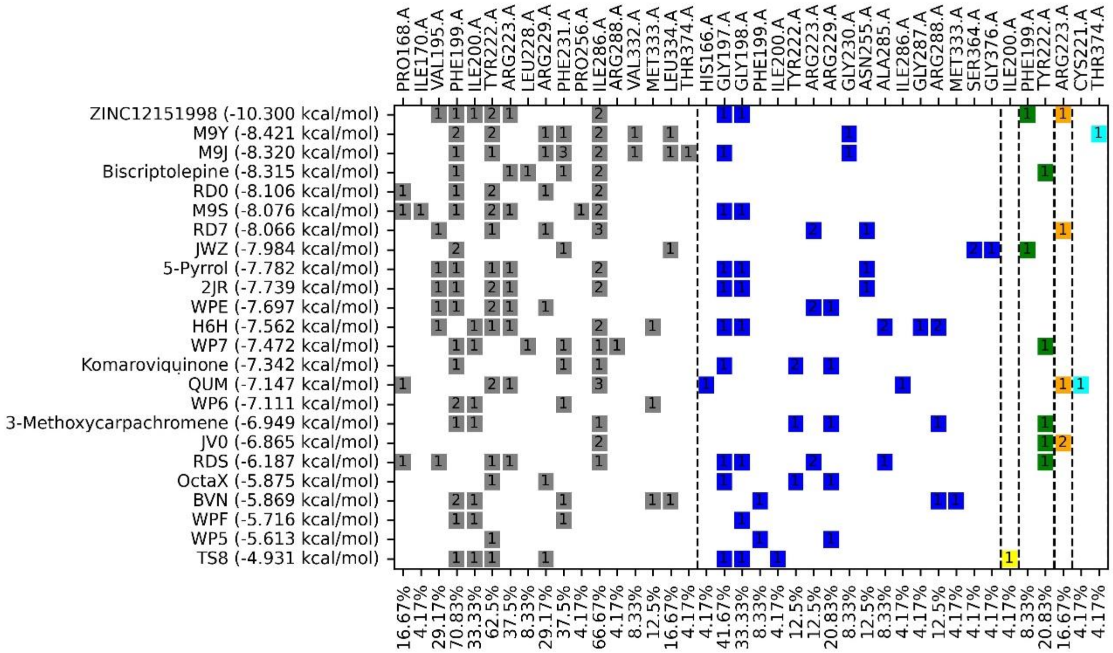

Mepacrine site

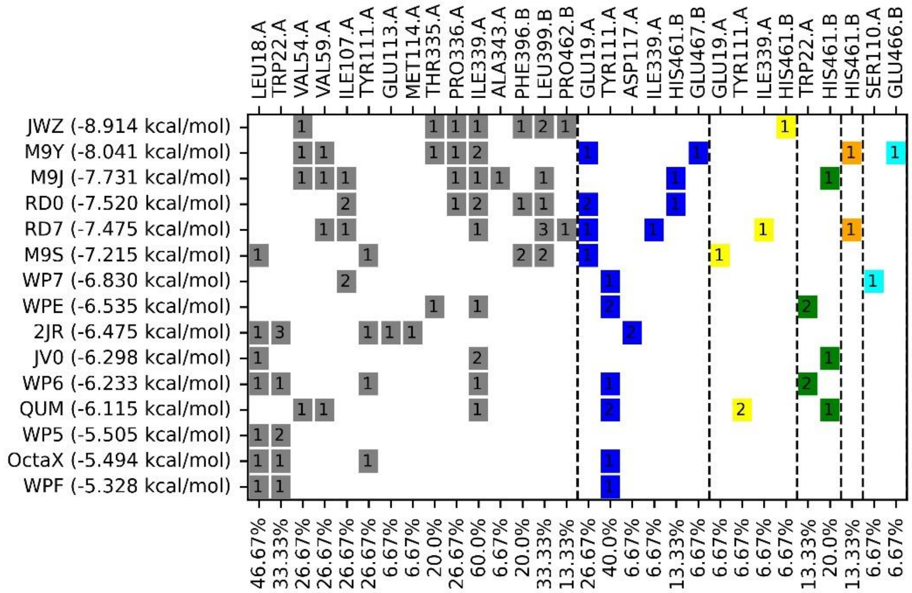

Catalytic site

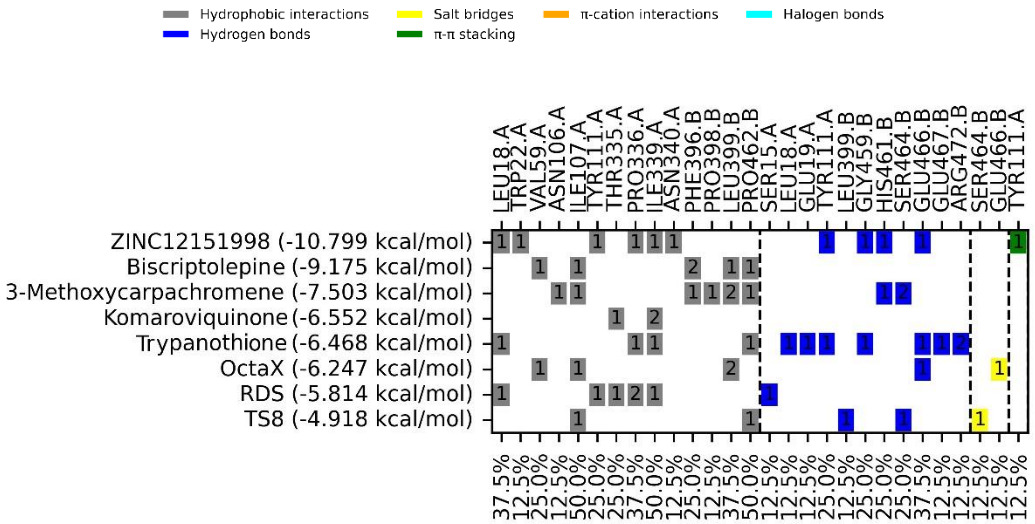

Supplement: Supplementary file 1 [file molecules-29-03796-s001.zip › molecules-3108904-supplementary.pdf]
